# Design and Development of Daily Morning Surgical Rounds in ICU by Quality Function Deployment

**DOI:** 10.1097/pq9.0000000000000171

**Published:** 2019-04-30

**Authors:** Sandeep Tripathi, Ann J. Naevor, LaMonica L. Henrekin, Karl F. Welke

**Affiliations:** From the * Department of Pediatrics, Children’s Hospital of Illinois, Peoria, Ill.; † Pediatric Critical Care Unit, Children’s Hospital of Illinois, Peoria, Ill.; ‡ Department of Surgery. Levine Children’s Hospital, Charlotte, N.C.

## Abstract

**Objective:**

Most healthcare quality improvement projects focus on change management rather than the design of the process. In this article, we describe the development of a new rounding model based on quality function deployment, a Design for Six Sigma tool.

**Methods:**

We used affinity diagram, focus groups, and Kano analysis to identify critical customer requirements. We also used Kano analysis to determine the design team’s ability to meet the requirements. We then completed a correlation matrix (House of Quality).

**Results:**

Fifteen quality characteristics were analyzed on a correlation matrix to meet 15 demanded qualities. Numerical values were calculated based on the interactions identified by the design team. We designed a new rounding model based on the House of Quality; this new rounding model resulted in higher provider satisfaction (median 76–80%, *P* = 0.06).

**Conclusions:**

We have described a sophisticated approach to the design of a new change strategy/intervention. This technique can be used as a starting point for other projects attempting to implement meaningful changes for complex processes of medical care.

## Abstract 2

Supplemental Digital Content is available in the text.

## INTRODUCTION

Most healthcare processes are complex with often conflicting requirements by different stakeholders. At a microsystem level, most process improvement steps rightly emphasize identification of a problem and data to measure change and its implementation (change management). However, as the science of quality improvement (QI) in health care has matured, there is now a need for more robust and scientific design processes. Only in the last few decades, service industries have started utilizing scientific methods for design. Design for Six Sigma (DFSS) is the branch of QI science that pertains to create a new product and process. One of the most frequently used tools in DFSS is quality function deployment (QFD).^1^

First described by Dr Yoji Akao for Japanese tire and ship manufacturing in the late 1960s, QFD was introduced to the United States in 1983 with primary work by Don Clausing of Xerox and later Massachusetts Institute of Technology and Bob King of GOAL/QPC.^2^ QFD in its essence guides development of the product’s technical requirements from the customer’s requirements; thus, it is possible for the design team to identify the attributes of a product, which would maximize customer satisfaction. The QFD process involves constructing a matrix (also called House of Quality; HOQ) displaying customer wants and needs (Voice of Customer) on the left and design team technical requirements to meet those needs on the top. Thus, QFD helps developers decide the relative importance of their choices by deriving their priorities from their customer’s priorities.

We conducted an online literature survey to identify prior published work on the utilization of principles of QFD in health care. The search included Medline, CINAHL, Med Journals@OVID, EBSCO academic, and PubMed. We used the search words “the use of QFD in healthcare.” The search resulted in 29 citations, of which only 8 reports were on improving services in health care.^3–10^ Although there are multiple published reports on improving communications in the intensive care unit (ICU) rounds,^11–13^ we did not find any report on the prior use of DFSS in ICU rounds’ improvement.

In this article, we describe the practical application and adaption of the QFD model for redesigning the pediatric cardiovascular ICU (CVICU) rounds. Although patients are the final customers (end users) of any medical process, for this project, the process of rounds is considered a “product” and the participants of rounds are considered as “customers.”

The CVICU at Children’s Hospital of Illinois (Peoria, IL) is an 8-bed unit, which is a part of 16-bed multidisciplinary pediatric ICU (PICU). The PICU admits about 1000 patients per year with approximately 50:50 medical/surgical split. Two cardiac surgeons operate on about 100–150 pump cases a year. CVICU rounds are conducted at the bedside with a collaborative team of surgeons, cardiologists, intensivist, pharmacist, and bedside nursing.

Our institution’s pediatric cardiac surgical rounds, thus, carried all the elements of a complex variable process including various stakeholders with different and often competing requirements. The complex process led to a high level of staff dissatisfaction and, in the subjective opinion of the providers, less than optimal patient care. In a recent (February to April 2016) internally designed staff satisfaction survey (n = 62), CVICU rounds scored a mean quality score of 60.9 (± 23.2), as opposed to an overall score of quality of care provided to patients in the PICU of 80 (± 15) on a scale of 1–100 with 100 the best possible quality (data not shown). The overall goal of this project was to design a rounding format with objective data based on customers’ demands, using market research and new product design tools adopted from industry.

## METHODS

**Fig. 1.**
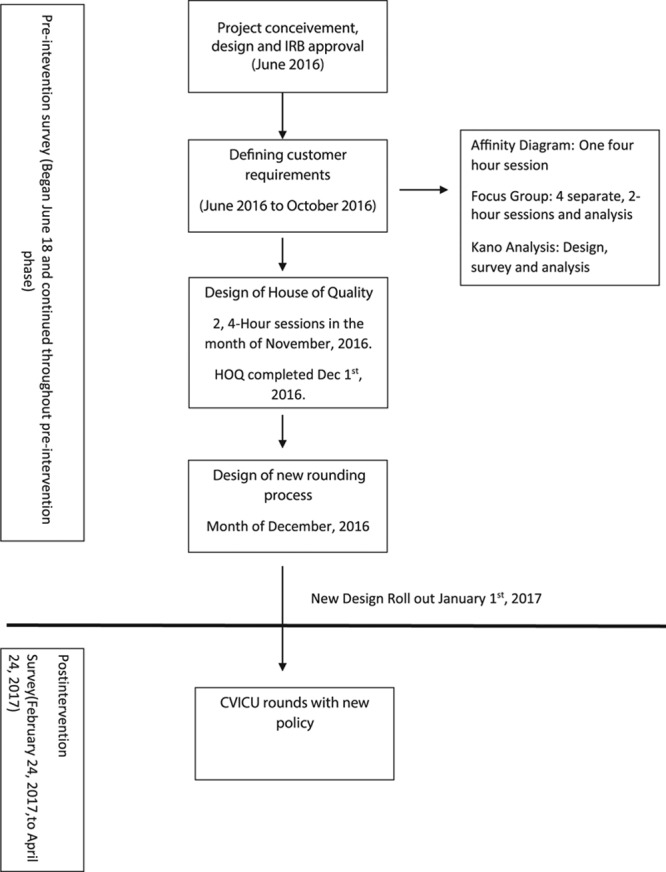
Project timeline and sequence of steps.

We established a dedicated cross-functional QFD team (including intensivist, cardiologist, surgeon, and nurse practitioners) with a mandate to design a rounding process with all stakeholders’ specifications and within the environmental and cultural limitations of the institution. The Peoria Institutional Review Board (IRB) reviewed the formal charter and deemed the project as QI. The project timeline and sequence of various steps are shown in Figure 1. Because with QFD most of the work is done in the design phase to minimize the rework, we did not undertake separate PDSA cycles in the deployment phase.

We used the following qualitative and quantitative market research tools in sequential order to assess the *critical to quality* customer requirements. The results of 1 tool was used as a basis for designing the questions for the next tool. The linkage between steps is not direct, and the design team used clinical judgment to tailor the results from 1 step into questions for the next step.

1. Affinity diagrams: after a brainstorming session, the design team along with the invited representatives of the various stakeholder groups created an affinity diagram to organize large numbers of ideas into their natural relationships. The team grouped all related ideas into groups and selected headings for the groups. The groups formed the basis of the questionnaire for the focus groups.
2. Focus groups: a trained facilitator who was not a primary member of the QFD team conducted 4 separate focus group sessions utilizing a preconstructed questionnaire. Focus group sessions were recorded, transcribed verbatim, and analyzed utilizing prior described methodology.^14^ The emerging themes from the focus groups were utilized to develop the Kano survey.
3. Kano analysis: Professor Noriaki Kano of Tokyo Rika University and colleagues first described the Kano survey in year 1984.^15^ They based it on the concept that not all customer requirements are created equal. Meeting some requirements can lead to heightened satisfaction but no dissatisfaction if these are not met (attractive requirements). For some other requirements, customers are not more satisfied if those are met. However, they are very dissatisfied if they are not met (mandatory requirements). Certain requirements are linear (performance requirements), as customer satisfaction behaves linearly to the presence or absence of this requirement (**see figure, Supplemental Digital Content 1** at http://links.lww.com/PQ9/A87; reproduced with permission.^16^) For this project, we created a 38-point Kano survey (16 presentation elements, 8 decision elements, and 14 process elements). We sought responses from all stakeholders. We then evaluated responses on a specially designed Kano matrix and analyzed them both qualitatively and quantitatively, to characterize into mandatory, attractive, performance, indifferent, and reverse categories by methods described earlier.^17^ We have published details of Kano analysis previously.^17^

### Development of HOQ

**Fig. 2.**
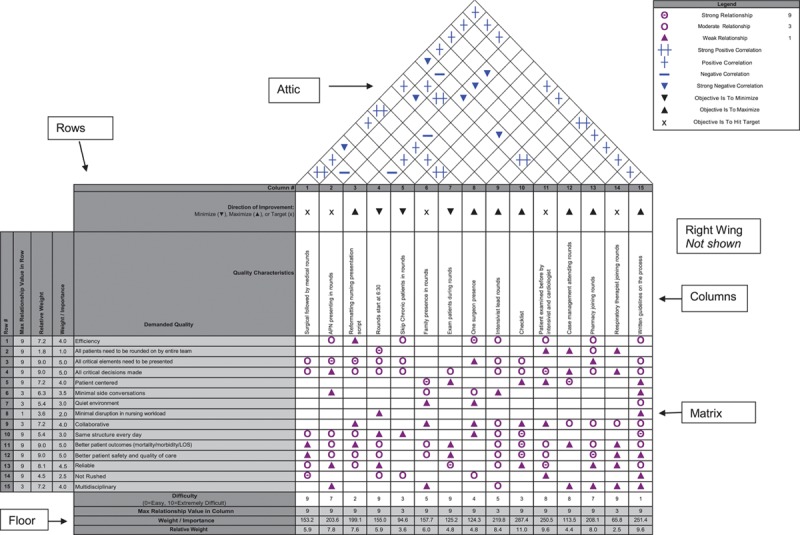
House of Quality (customer-demanded quality as rows and design team’s options as columns). Matrix showing interrelationships, depicted by symbols. Legend box shows values of the symbols. Refer to the text for description.

After evaluating customers’ requirements and potential service offering/practices that meet these requirements, we constructed a HOQ on a template available for free download on the Internet^18^ in the following steps (Fig. 2):

Step 1: The row (customer wants or “what’s”): the QFD team selected *demanded qualities* based on prior described customer needs’ assessment methods (affinity diagrams, focus group, and Kano analysis). The team then entered weights (importance) in a column to the left of the row labels on a scale of 1–10, where 10 is extremely important and 1 is unimportant. The QFD team assigned these weights based on information obtained from the customers.
Step 2: The columns (quality attributes of the product or service or “how’s”): we based these high-level descriptions of planned services primarily on Kano process questions. The QFD team added a row between the *how’s* and the *attic* with symbols indicating whether the characteristic needs to increase or decrease to meet better customer’s requirements (we based this directionality on Kano categories).
Step 3: The matrix (relationship among the rows and columns): the interaction between rows and columns is the primary and most important aspect of the HOQ. These values were selected based on the development team’s judgment of the strength of the relationship between each element of their technical response and each customer’s wants and needs.
Step 4: The roof or “attic” (implementation interrelationship between elements of the technical response): this part of the HOQ reveals tradeoffs between various design options that have to be considered. Symbols were used to indicate that the relationship is +ve or −ve and how strong it is.
Step 5: The right wing (customers’ perception of existing comparable services by competitors): this section did not apply to our HOQ, as we are the only CVICU in the region.
Step 6: The floor (weights and relative importance): are created by multiplying relationship number by the importance of that customer requirements. We then added the results for the entire column. This sum is the weight of that characteristic or computed rank ordering of the technical response. A row is added to include technical difficulty in accomplishing that design element. We assign this weight on a scale of 1–10, where 10 would be the hardest thing to change, based on local culture and resources.

### Satisfaction Survey

To monitor and compare the satisfaction, we conducted a 6-element (content, communication, collaboration, environment, engagement, and time) rounds’ satisfaction survey on random days throughout the project. We allowed about 2 months after implementation to allow for teams familiarity with the process. The preimplementation survey was done over 5 months, whereas postsurvey was done over 2 months (February 24, 2017, to April 24, 2017) (Fig. 1). We graded all the responses with content, communication, and collaboration on a scale of 1–5, environment and engagement on a scale of 1–4, and time on a binary scale. We calculated the final satisfaction score by dividing the total score across all domains by the maximal possible score of 25. With this model, we gave the highest weight (20%) to content, communication, and collaboration and the lowest weight to the time of rounds (8%). We gave this weight based on the value perceived by the team. Questions were written so that the final average weight would account for the value. We compared the satisfaction score before and after the process change to assess the impact of the new design. Test of normality by Shapiro–Wilk *W* test showed that data were not normally distributed (*P* = 0.02), so a nonparametric Wilcoxon rank sum test was used to compare the median of satisfaction surveys. We performed all statistical analysis by JMP statistical software (SAS Inc, NC). We present data as median [interquartile range (IQR) 25–75%].

## RESULTS

### Affinity Diagrams

Participants were asked to write on sticky notes the rounding processes we need to start, stop, or continue doing. We grouped these responses into similar categories. At the end of the session, we identified 22 processes to continue, 32 processes to start, and 17 processes to stop. We grouped these actions into 20 themes (7 to continue, 6 to start, and 7 to stop) (**see Supplemental Digital Content 2** at http://links.lww.com/PQ9/A88).

### Focus Groups

To further explore the actions identified in the affinity mapping, we developed a questionnaire with 3 engagement questions (based on “start,”, “stop,” and “continue” themes from affinity diagram) and 7 explorative questions. We coded a total of 250 comments based on 8 emerging themes [satisfaction 8.8%, process 9.2%, structure (who 6.8%, what 41.2%, when 8%), leadership 6%, engagement 7.2%, environment 3.6%, relationship 3.2%, and goal 6%]. The analysis was done separately for the 4 focus groups of the rounding team (nurse, intensivist and nurse practitioners, cardiologist, and surgeons) and cumulatively for all the responses in the 4 sections (**see Supplemental Digital Content 3** at http://links.lww.com/PQ9/A89).

### Kano Analysis

Twenty-eight providers completed the Kano survey (nurses 8, nurse practitioners 5, intensivists 6, surgeons 2, and cardiologists 7). In the presentation and decision category, respondents identified only 1 element as attractive (1-line statement about the patient), whereas they categorized 6 process elements as attractive. There were 5 mandatory presentation and 3 mandatory decision elements, but customers of the rounding process considered no process elements as mandatory. We scored 3 process elements as the reverse (“rounds to start at 6:30 am in the morning,” “skip chronic patients on surgical rounds,” and “exam patients during rounds”). We have discussed mathematical categorization and ranking of these elements elsewhere (Table 1).^17^

**Table 1.**
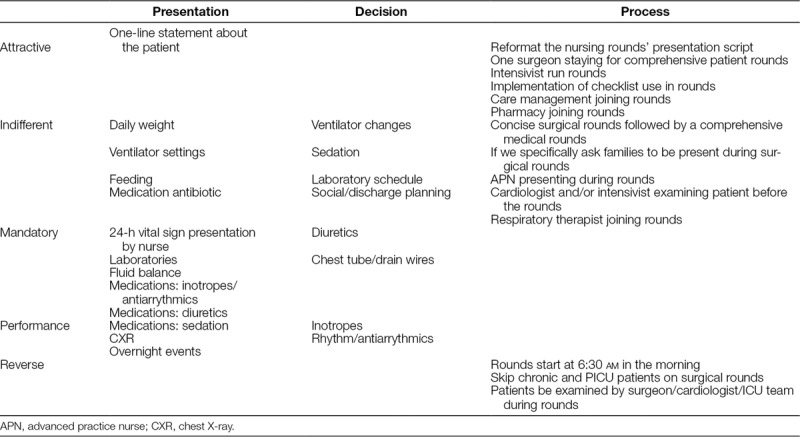
Results of Kano Analysis

### Quality Function Deployment

We listed the 15 demanded quality measures in rows and assigned them weights based on the consensus of the QFD team. The team gave the maximum weight of 5 to “better patient safety and quality of care,” “better patient outcomes,” “all critical elements presented,” and “all critical decisions made.” They gave the least weight of 1 to “all patients need to be rounded by the entire team.” We calculated the relative weights of each demanded quality by dividing the individual weight with the sum of the total weights in the column and expressed it as a percentage. We listed 15 quality characteristics in columns. Seven characteristics were to increase for improvement, 3 characteristics were to decrease, and 5 characteristics had no directionality. The 3 characteristics targeted to decrease (“rounds start at 6:30 am in the morning,” “skip chronic patients on rounds,” and exam patients during rounds) were identified as the reverse category on the Kano analysis. After completion of the interrelation matrix, the quality characteristics that had the highest impact on the demanded quality were “checklist” (287.4), “written guidelines on the process” (251.4), and “patient examined before by the intensivist and cardiologist” (250.5). The characteristics with the lowest impact were “respiratory therapists joining rounds,” skip chronic patients on rounds (94.6), and “care management joining rounds” (113.5). Similar to the relative weights on demanded quality, we also calculated the relative weights of the quality characteristics. The design team then ascribed the difficulty rating with 9 (most difficult) given to 4 categories and 1 (easy to accomplish) to 1 category. We created the correlation matrix at the end with 36 total possible interactions with 6 at 2+ and 19 at 1+ with 7 strong negative and 4 negative interactions. The interactions were specific to local culture and practices and may be different at other facilities (Fig. 2).

### Satisfaction Surveys

We completed a total of 81 surveys in the baseline phase (nurse practitioners 15, cardiologists 4, intensivists 15, nurses 41, pharmacists 3, and surgeons 3) and 31 surveys (nurse practitioners 7, cardiologists 1, intensivists 5, nurses 16, pharmacist 1, and surgeon 1) in the postintervention phase. The overall satisfaction score showed improvement but did not reach statistical significance [76% (IQR 60–84) versus 80% (IQR 72–88), *P* = 0.06]. Because nursing and medical providers may have different perceptions of the rounding process, these scores were also evaluated separately across all domains. Nursing providers had much higher median satisfaction scores; however, overall improvement in nursing satisfaction did not reach statistical significance [80% (IQR 74–84) versus 84% (IQR 77–96), *P* = 0.10]. Among different domains, there was a statistically significant difference among medical providers’ perception of improved information transfer process [median score 2 (IQR 2–3.75) versus 3 (IQR 2–4), *P* = 0.04], whereas there was a significant difference in the nursing perception of improvement in critical decision making [median score 4 (IQR 3–4.5) versus 5.5 (IQR 4–5), *P* = 0.04]. The difference in other domains was insignificant for both nursing and medical providers (Table 2).

**Table 2.**
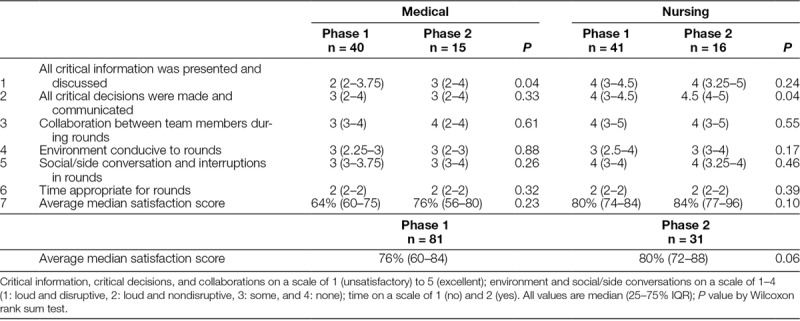
Satisfaction Survey Comparison in the 2 Phases

## DISCUSSION

**Fig. 3.**
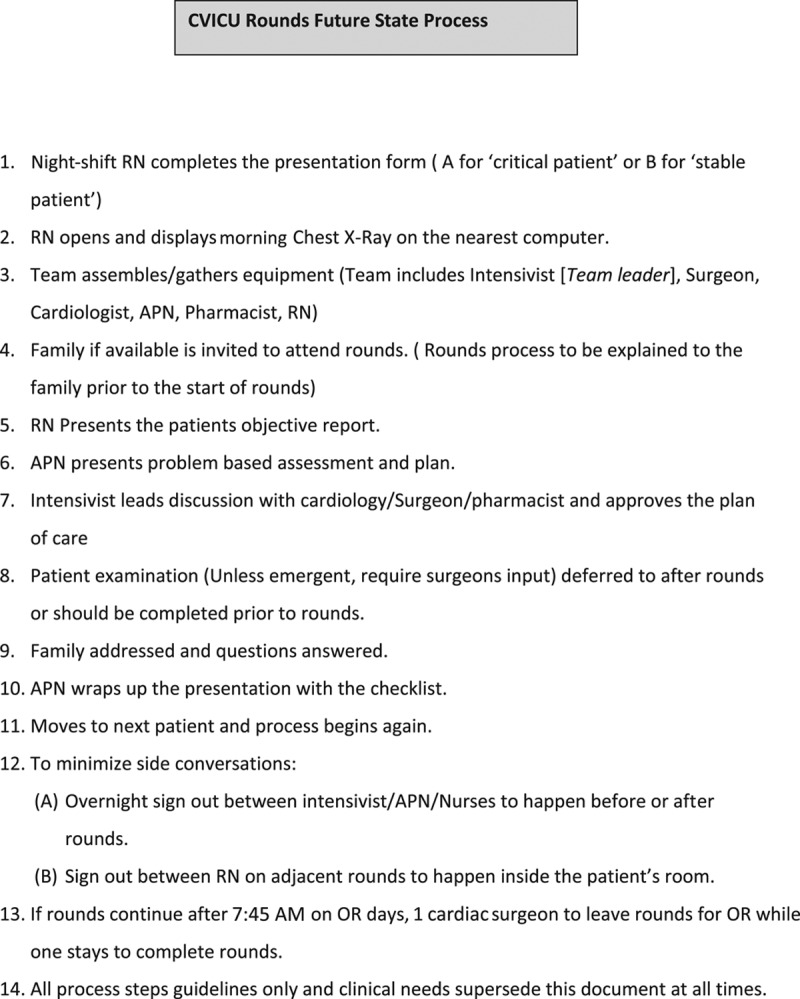
Future state process. APN, advanced practice nurse; RN, registered nurse; OR, operating room.

This article describes the successful adaptation of QFD in the design of the ICU rounding process. Based on the results of this process, we devised and implemented a new rounding policy (Fig. 3). Although this process leads to an improvement in satisfaction scores, the difference (pre–post) was not statistically significant. Although QFD has been described earlier,^4–6,17^ most of the prior work has been done on new material, equipment, or facilities design. Ours is the first reported process to describe its utilization in a multidisciplinary clinical process like rounds.

Our project combined the techniques of Kano and QFD. Because both of these tools focus on customer needs and service attributes, this combination yields a very powerful and reliable design template. Matzler and Hinterhuber^19^ proposed this concept in 1998. Kuo et al^10^ were the first investigators to demonstrate practical utilization of a combined Kano and QFD method. They utilized QFD and Kano methods to evaluate and improve the quality of outpatient services for elderly patients. Using the Kano model and an integrated analytic network process-QFD approach, they extracted 5 needs of elderly patients and their priorities. They also identified 6 outpatient services attributes deserving of improvement and their priorities. The integrated QFD model thus created revealed the crucial outpatient service items. We adopted a methodology similar to Kuo et al^10^; however, we did not use the exact coefficients of the various process, decision, and presentation elements of QFD. In our situation of many conflicting requirements of equal priorities, we believed that a strictly mathematical approach would have led to an unreasonable limitation to the design team. Although not precisely analytical, it allowed the design team some latitude when faced with mutually interdependent attributes. Our intent in this article was to describe the principles of QFD with a practical application. Different hospitals would have varying quality requirements, and our HOQ is not intended to apply to other units.

### Limitations

The design team did the ranking metric by consensus. Due to different disciplines of stakeholders (customers), even a perfectly designed product will leave some dissatisfied customers, as is evident from our postsatisfaction data. Although subjective, the consensus was an informed decision based on multiple customer feedback methods chosen. A more scientific consensus building technique (like Delphi method) would have made HOQ more robust.

Similarly, Kano analysis was done but not directly linked to the *what’s* and how’s, also leaving the possibility of bias by the design team itself. Our project did not include true customers’ (patients and families) opinions for the lack of resources. This exclusion is a significant omission in our design, and our next HOQ design will include patients/families as stakeholders. Our study also did not show a significant increase in customer satisfaction. We believe that the baseline scores were higher than expected due to the Hawthorne effect (practice had already started to shift in the preintervention phases because the staff was aware of observation and monitoring), thus affecting improvement in the postimplementation phase. Lastly, although Kano and HOQ are robust tools, they require additional time and resources, which may be challenging for many pediatric centers trying to engage in local QI work with limited expertise in these methodologies.

## CONCLUSIONS

A complex multivariable process with stakeholders of varying interests and requirements like ICU rounding can be designed and implemented successfully with HOQ. This technique may be a starting point for projects trying to accomplish meaningful changes to this most ingrained and complex processes of medical care.

## DISCLOSURE:

The authors have no financial interest to declare in relation to the content of this article.

## Supplementary Material
