# Supplementary figures and images for: Design and Development of Daily Morning Surgical Rounds in ICU by Quality Function Deployment
Source: Pediatr Qual Saf. 2019 Apr 30;4(3):e171. doi: 10.1097/pq9.0000000000000171 (PMC6594777; doi:10.1097/pq9.0000000000000171)

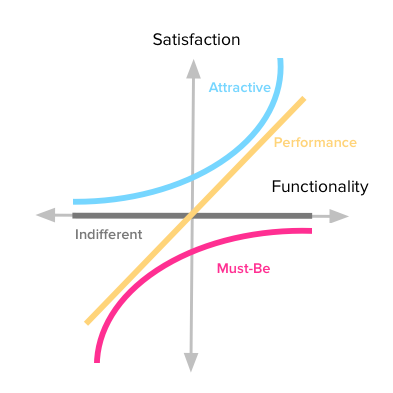

Supplement: Supplementary file 1 [file pqs-4-e171-s001.tif]
