# Supplementary material for: Design and Development of Daily Morning Surgical Rounds in ICU by Quality Function Deployment
Source: Pediatr Qual Saf. 2019 Apr 30;4(3):e171. doi: 10.1097/pq9.0000000000000171 (PMC6594777; doi:10.1097/pq9.0000000000000171)
Supplement: Supplementary file 2 [file pqs-4-e171-s002.docx]

**Interdisciplinary**

START

Start doing rounds the same way

Start on time

Start rounds sooner. On time. No matter who is there.

A systematic way to facilitate rounding time

Start consistently writing orders during rounds

Consistent exams before decisions are made

Consistent order placement

Timely

**Structure**

Discussion and plan for each major problem/system on each patient after RN presentation and examining patient

Pre organization of data being presented to make a more comprehensive picture

Start with concise summary of overnight clinical course

ABG presented

Efficient presentation from log or check list

All data presented one time

Examine at the time of discussion and decisions

Review events from overnight before RN presentation

Identify critical information

Examine patients during rounds to facilitate best plan of action

Clear plan of day

Orders written

Pacemaker check during rounds

APN presenting

RT scheduled

**Culture**

Culture of gratuity and teamwork

Surgeons present or not present: Just make a decision

Everyone engaged

Mutual respect for co-workers and their thoughts

Preparedness

**Time**

Start rounds at 6:30

Start earlier 6:45

**Teaching**

Education tidbit to nurse

**Family**

Family involvement

**Interruption**

STOP

Interrupting a person’s thoughts: over talking

Interrupting nurses in report

Sign out occurring in rounds

Distractions and social talk

Stop conversations between the groups

Slow transition between patients

**Lack of Family**

Stop excluding families

**Decision Paralysis**

Procrastinating therapies “we will look into that and we don’t for weeks”

**Long Teaching**

Lengthy teaching sessions as bedside

**Rushing**

Rushing and moving next patient before discussion on prior patient is finished

**Repetition**

Repetitive information

Double rounding

Reading normal labs

Daily rounds on chronic patients by surgeons

**Fixed Time**

Having a stop time to rounds

**Interdisciplinary**

CONTINUE

Interdisciplinary team

Need to continue all disciplines present

Comprehensive rounds with multidisciplinary

All team members present during rounds on all critical patients

Team collaboration: every team member’s perspective

**Nursing**

Continue nurse presentation of data

Preferably night shift nurse to present

Nurse involved in presenting

All elements of current RN presentation

**Culture**

Respect one person speaking at a time

All members of the team engaged

**Structure**

Same time everyday

Pharmacy included

Orders written during rounds

Pertinent data / information / CXR

**X-Rays**

Reviewing X-rays on each patient

Nurse has x-ray ready

Look at x-ray

**Plan of Care**

Clear plan of care for the day

Recap plan of care

**Family**

Family involvement
