# Supplementary material for: Design and Development of Daily Morning Surgical Rounds in ICU by Quality Function Deployment
Source: Pediatr Qual Saf. 2019 Apr 30;4(3):e171. doi: 10.1097/pq9.0000000000000171 (PMC6594777; doi:10.1097/pq9.0000000000000171)
Supplement: Supplementary file 3 [file pqs-4-e171-s003.docx]

**Cardiac Rounds Redesign Workgroup (CR^2^EW) Focus Group Questions**

Moderator: Kaila Dunnuck

Assistant Moderator/Recorder: Deanna Marie Klumb

**Opening statement**: This focus group is a part of a series of methods we are utilizing to get stakeholders input as we redesign the rounding process. Today’s focus group discussion will be recorded to obtain detailed minutes, however, the recording will be deleted as soon as minutes are obtained & no names will be included in the minutes. (1 Minute)

**Introductory question**: Can you identify yourself and how many years of experience do you have with rounding on post-operative cardiac patients in the ICU. (2 Minutes)

**Transition question**: What is one thing that comes to mind while describing our cardiac CV rounds? (2 minutes)

**Key Questions**

1. Engagement question: Can you list some of the activities that we should start, stop or continue doing? (5 Minutes)

*Exploration question: What do you like the best about the rounds?*

*What do you like least about the rounds?*

1. Engagement Question: The workgroup has compiled a list of elements that can be presented/discussed and/or decided during rounds. When you think of these categories, do any other needs come to mind? (7 Minutes)

*Exploration Question: Of all the things we discussed what is most important to you?*

*What is the most critical element that should be discussed during rounds?*

1. Engagement Question: As we work on redesigning rounds, what do you think we can do differently to improve our practice? (15 Minutes)

*Exploration Question: What can be changed about how we do our Rounds (For ex, start rounding early) [Clarification Statement]*

*Can anyone present a different point of view?*

*What are the pros and cons of different approaches?*

**Ending question**: Is there anything we should have talked about but did not? (2 minutes)
